# Supplementary material for: The possibilities of the use of N-of-1 and do-it-yourself trials in nutritional research
Source: PLoS One. 2020 May 6;15(5):e0232680. doi: 10.1371/journal.pone.0232680 (PMC7202616; doi:10.1371/journal.pone.0232680)
Supplement: Attachment — Submitted filename: Rebuttal letter.docx [file pone.0232680.s003.docx]

Reply to the reviewers:

Both reviewers made excellent contributions, that we used in our full revision of the paper.

One of the first points of reviewer one, was the short introduction into the literature. We extended the introduction, but do not believe this should be the focus of the current paper. As such we refer to other papers which discuss the methods more extensively.

Further on, reviewer one mentions the fact that there is no autocorrelation and time trends assumed and that we have assumed stationarity. This is correct: to keep the model simple and focus on showing the difference between control and treatment, we decided on this course. We understood that this was not eloquently enough mentioned, as such we explicitly state our acceptance of this in lines 315-319 and 377-392.

Reviewer 2 mentioned that the goal of the research was not clear enough in our paper, we explicitly stated this now in 109-111.

Furthermore, they pointed out that our models were not described sufficiently, in this new version we described the models in 114 to 156. This is not a fully, technical description, as the models are fairly common and more extensively described in the references mentioned. The model used for simulation, which is based on these models, is noted in 168-195.

Thirdly, they placed question marks with our choice to select only two methods for the simulation study. We fully agreed with these questions, and put all three methods in the new simulation study. With this, we also discussed the needed and used fit-measures and rewrote both methods and results, dealing with points 5, 6 and 7 of reviewer 2.
