# Supplementary material for: The possibilities of the use of N-of-1 and do-it-yourself trials in nutritional research
Source: PLoS One. 2020 May 6;15(5):e0232680. doi: 10.1371/journal.pone.0232680 (PMC7202616; doi:10.1371/journal.pone.0232680)
Supplement: Attachment — Submitted filename: Response to Reviewers.docx [file pone.0232680.s004.docx]

**Additional Editor Comments**

Major issues:

1) "Meta-analysis" is a broad term that covers any type of statistical combination of studies; so ALL the methods you include are meta-analyses. You seem to be confusing "study-level" (or "aggregate data") meta-analysis with "individual participant data (IPD)" meta-analysis (NOTE the term "individual-subject" is not generally used).

*We adjusted the terms as requested.*

2) IPD meta-analysis generally uses mixed effects modeling, so your terminology is confused. As such I would not recommend study-level meta-analysis of N of 1 trials (except in cases where the participant-level data is unavailable)

*In general, mixed effect models analyze the data as a whole, allowing for weights, but do not use the two-step method as proposed. We clarified our explanation to make this clear.*

3) "Study-level" meta-analysis does not assume all individuals (in N of 1 trials) have the same effect. This is clear if you read DerSimonian and Laird.

*We adjusted our explanation.*

*Points 1, 2 and 3 are taken into account in the revision of the explanation in lines 122-166.*

4) Zucker et al consider a much more detailed range of models than your paper. This includes various random-effects correlation structures, and a range of Bayesian priors. You must either consider these various models, or make clear why you are using a more restricted choice.

*We choose to vary the dataset instead of the models, to show the effect of the different data on the results. We hope this is clearer now, see lines 230-237.*

5) Given the above points, you must set out the models your are proposing with much greater detail and clarity than in the current paper.

*We hope clarity is added by the numerous changes we made throughout the paper, clarifying the model, the data generation, the differences we propose to address and the results.*

6) It isn't clear to me how you simulated studies, but I assume a linear mixed effects structure was used. Obviously then, any mixed effect modeling that matches this structure will give the best results. You should be careful that your conclusions are not a consequence of how data were simulated.

*The simulation of the studies is described in lines 230-256. We explicitly stated how we simulated the data step by step, while following the equations 1 through 4. In lines 170-181, the rationale behind this was stated.*

**Reviewer #1**

Thank you for the opportunity to review The Possibilities of the use of N-of-1 and do-it-yourself trials in nutritional research. I enjoyed reading the manuscript, and believe it will make a valuable contribution. However, I do have a few concerns that I will list in the remainder of this review.

1. Although one way of conducting an ISMA is to use the model defined in Equation 1 assuming homogeneous treatment effects, I think other models and assumptions are also possible. Similarly, mixed effect modeling allows for a variety of models, assumptions, and estimation methods. In the discussion (see lines 377 to 385) the authors do a good job of acknowledging that model assumptions may be violated and more complex models could be estimated. It may be helpful to acknowledge some of these complexities in the initial presentation of the three approaches, so that it doesn’t appear that there are three specific models, but rather there are three methodological approaches, each with potential variations.

*Thank you for your kind words. We hope to have been more clear about the models in the rephrasing of the model section, and rephrased the comparison as being between the one-step or two-step approach.*

2. Related to Point 1, I found myself wanting a rationale for why the specific model shown in Equations 1 through 4 was chosen for the simulation. I think it was a reasonable choice, but also think it is helpful to be transparent about when and why choices are made.

*We added a rationale, indicating for each point why it was included, in lines 170-181.*

3. It would be helpful to formally define the meta-analytic model that was used to synthesize the effect sizes for the ISMA.

*We hope to have done this in lines 205-211.*

4. Also related to Points 1, 2, and 3, I found myself wondering how much of the difference in the simulation results can be attributed to the methodological approach (2 steps where effects were first estimated and then meta-analyzed) versus to the difference in assumptions (e.g., homogeneity of treatment effects versus heterogeneity of treatment effects)?

*As we adjusted our explanation and rationale, we now believe that the two – step approach and different approach of obtaining the effect, e.g., assuming a distribution as opposed to taking the mean, are of influence here. We hope to be clear about this in lines 205-211 and 284-288. While an exact study on how these difference come to pass and which is due to what would be highly informative, it is beyond the scope of the current paper. To address this fully, we would have to simulate data with related properties.*

5. On line 90 should “The most important different” be “The most important difference”?

*Yes, you are quite right, we adjusted this. Thank you.*
